# Totally laparoscopic verse laparoscopic assisted distal gastrostomy for gastric cancer: an update meta-analysis

**DOI:** 10.18632/oncotarget.23895

**Published:** 2018-01-03

**Authors:** Borong Chen, Disheng Xiong, Zirong Pan, Mingyuan Chen, Gang Liu, Shengjie Wang, Yongzhi Ye, Rui Xiao, Junjie Zeng, Jiayi Li, Zhengjie Huang

**Affiliations:** ^1^ Department of Gastrointestinal Surgery, Xiamen Cancer Hospital, First Affiliated Hospital of Xiamen University, Xiamen, China; ^2^ Department of Gastrointestinal Surgery, First Clinical Medical College of Fujian Medical University, Fuzhou, China; ^3^ Department of General Surgery, Xiamen Haicang Hospital, Xiamen, China; ^4^ Department of Hepatobiliary Surgery, Fujian Medical University Union Hospital, Fuzhou, China; ^5^ Department of Medical Oncology, Xiamen Cancer Hospital, First Affiliated Hospital of Xiamen University, Xiamen, China

**Keywords:** laparoscopic gastrostomy, gastric cancer, meta-analysis

## Abstract

Totally laparoscopic distal gastrostomy (TLDG) and laparoscopic- assisted distal gastrostomy (LADG) are the minimally invasive surgical technology for gastric cancer. This study aimed to compare the surgical outcomes of these two methods. Relevant studies were selected through electronic searches of EMBASE, PubMed and Web of Science. In total, 21 non-randomized controlled studies containing 2475 patients in the totally laparoscopic distal gastrostomy and 1889 patients in the laparoscopic-assisted distal gastrostomy were included in this study. And operative time, operative blood loss, retrieved lymph nodes, time to liquid diet (days), postoperative hospital stay and overall complications were pooled and compared using meta-analysis. There were no significant differences between operative time (WMD = 0.38, 95% CI –10.43 –11.18, *P =* 0.95) and overall complications (RR = 1.09, 95% CI 0.91–1.30, *P =* 0.36). But totally laparoscopic distal gastrostomy had more advantages in aspects of intraoperative blood loss (WMD = 24.4, 95% CI 12.45–36.36, *P* < 0.0001), time to liquid diet (days) (WMD = 0.21, 95% CI 0.03–0.40, *P =* 0.03) and postoperative hospital stay (WMD = 0.72, 95% CI 0.31–1.13, *P =* 0.0006). Moreover, totally laparoscopic distal gastrostomy had more retrieved lymph nodes (WMD = –1.24, 95% CI–1.90 to–0.58, *P =* 0.0002). This meta-analysis indicates that totally laparoscopic distal gastrostomy may be a safe, feasible, and favorable surgical technology in terms of less blood loss, faster liquid diet, shorter postoperative hospital stay and more lymph nodes retrieved.

## INTRODUCTION

Gastric cancer is one of mostly common digestive tract tumors and the second leading cause of cancer death with worldwide distribution [1]. Since the introduction of laparoscopic gastrostomy (LG) in 1994 [2], LADG for gastric cancer has undergone rapid development and widely accepted in clinical practice as a result of improvements in surgical techniques and devices in the past 20 years. Especially in Japan, Korea and China, based on a number of reports that have presented the benefits of LADG, compared with conventional open gastrostomy, LADG was acknowledged as having advantages such as less blood loss, faster recovery, fewer postoperative complications, less pain, shorter hospital stay, more desirable cosmetic result and better postoperative quality of life [3–5].

TLDG is considered ‘incisionless’, except for the trocar wounds, and it is a laparoscopic approach for intracorporeal anastomosis without auxiliary incision [4]. TLDG preserves the integrity of the abdominal wall, therefore, TLDG is considered less operative trauma, better recovery and cosmesis can be expected [6]. Some articles has reported that TLDG is considered less invasive than LADG [7]. However, there have been few prospective studies of differences in the clinical results of TLDG and LADG. Some surgeons deem that LADG is more preferable than TLDG, because of difficulties in performing an intracorporeal anastomosis and limited experience. Given these reasons, we compared the surgical-related outcomes of patients treated with TLDG and the patients treated with LADG. A systematic review with meta-analysis was conducted to further clarify the safety and feasibility of TLDG. Surgically-related results are discussed according to the best scientific literature available.

## RESULTS

### Study characteristics

**Figure 1:**
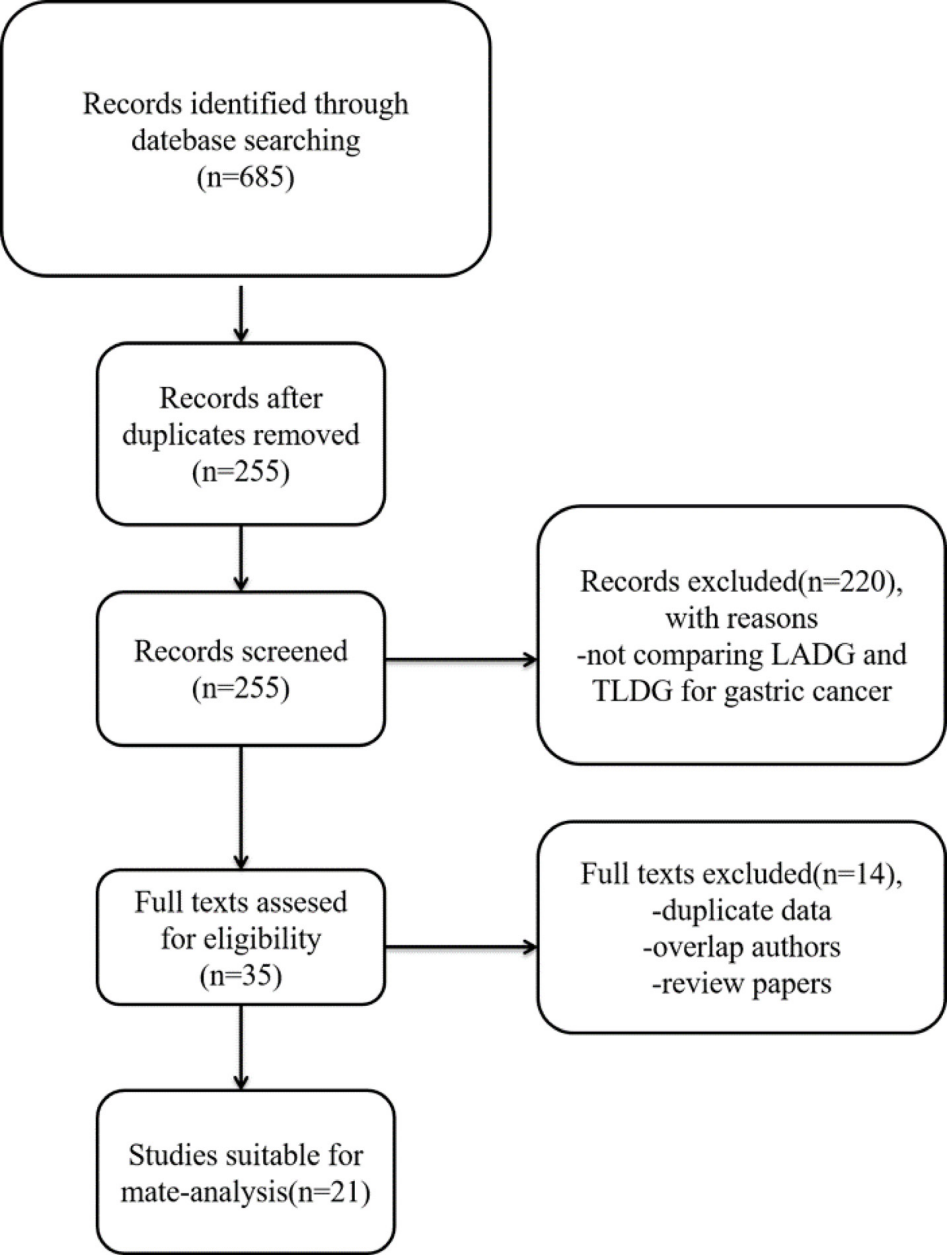
Flow chart of the selecting process of literature

685 relevant studies were extracted initially. The literature selection process was shown in Figure 1. Finally, a total of 21 studies that were published between January 2008 and June 2017 that matched the selection criteria and were therefore included [8–28], and they comprised 4364 patients in total, 2475 of whom underwent LADG and 1889 of whom underwent TLDG. The major baseline characteristics of the 21 eligible publications were reported in Table 1.

**Table 1:** The characteristics of the included studies

References	Country	Operation types		Sex of patients (M/F)		Age of patients (M ± SD)		Level of lymphadenectomy	Reconstruction
		LADG	TLDG	LADG	TLDG	LADG	TLDG		
Song (2008)8	Korea	20	20	12/8	13/7	58.5 ± 10.1	56.7 ± 13.5	D1 + β, D2	B-I, B-II, R-Y
Ikeda (2009)	Japan	24	56	16/8	28/28	64.5 ± 11.9	63.5 ± 11.2	D1 + β, D2	B-I, R-Y
Kim MG (2010)	Korea	328	239	198/130	155/84	55.4 ± 11.2	56.6 ± 12	D2	B-I
Kinoshita (2011)	Japan	41	42	30/11	25/17	68.4 ± 10.3	64.7 ± 10	D1 + α/β, D2	B-I
Lee J (2012)	Korea	269	130	161/108	75/54	62.5 ± 12.0	61.0 ± 11.8	D1 + α/β, D2	B-II
Choi (2013)	Korea	35	37	22/13	23/14	67.9 ± 10.1	65.2 ± 10.9	D1 + α/β, D2	B-I, B-II, R-Y
Kim DG (2013)	Korea	106	60	69/37	37/23	55.8 ± 12.5	58.3 ± 12.5	D1 + β, D2	B-I
Kim HG (2013)	Korea	136	111	91/45	77/44	60.1 ± 11.7	61.0 ± 11.2	D1 + β, D2	B-I, B-II
Chen K (2014)	China	93	147	NR	NR	NR	NR	D2	B-I, B-II
Han (2014)	Korea	77	134	49/28	77/57	58.2 ± 10.4	57.2 ± 12.7	D2	B-II
Kanaji (2014)	Japan	74	40	51/23	23/17	66 ± 9	63 ± 12	D1 + α/β, D2	B-I, B-II, R-Y
Lee SH (2015)	Korea	99	33	58/41	20/13	58.8 ± 11.6	58.5 ± 12.2	D1 + α/β, D2	B-I, B-II, R-Y
Kim SM (2015)	Korea	100	102	50/50	63/39	50 (32–75)	52 (29–84)	D1 + β, D2	B-I, B-II, R-Y
Woo (2015)	Korea	55	55	37/18	35/20	59.0 ± 10.7	61.3 ± 11.9	D2	B-I, B-II, R-Y
Zhang B (2015)	China	45	24	31/14	16/8	NR	NR	D2	B-I, B-II, R-Y
Zhang C (2015)	China	25	11	7/4	16/9	62.24 ± 2.375	63.64 ± 2.516	D1 + α/β, D2	B-I, B-II, R-Y
Chen K (2016)	China	145	108	98/47	73/35	57.3 ± 12.5	59.4 ± 11.1	D1 + α/β, D2	B-I, R-Y
Nishimura (2016)	Japan	69	126	44/25	87/39	60.1 ± 11.7	61.0 ± 11.2	D2	B-I, B-II, R-Y
Shinohara (2016)	Japan	43	57	25/18	36/21	72 (40–86)	70 (38–80)	D1 + α/β, D2	B-I, B-II, R-Y
Lin M (2016)-1	China	484	158	337/147	102/56	59.9 ± 11.7	59.0 ± 13.1	D1 + α/β, D2	B-I
Lin M (2016)-2	China	143	143	102/41	100/43	59.4 ± 12.1	60.1 ± 12.7	D1 + α/β, D2	B-I
Kim JH (2017)	Korea	60	60	40/2	40/20	60.9 ± 11.4	60.5 ± 12.1	D1 + α/β, D2	B-I, B-II, R-Y

TLDG: Totally laparoscopic distal gastrostomy; LADG: laparoscopic-assisted distal gastrostomy; B-I: Billroth I; B-II: Billroth II; R-Y: Roux-en-Y; NR: not reported.

### Meta-analysis results

#### Operating time

**Figure 2:**
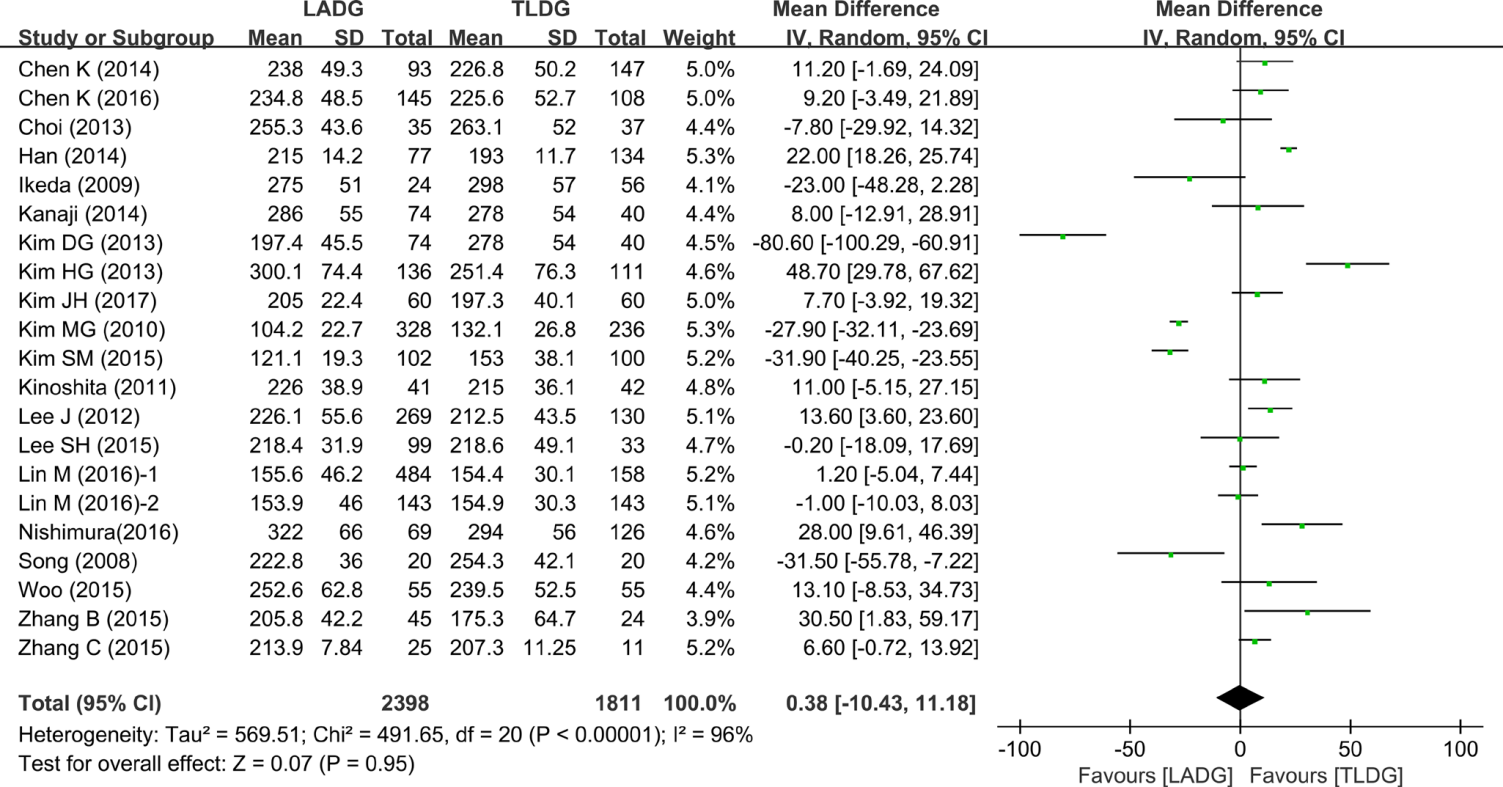
Meta-analysis of the pooled data: operation time

The results of meta-analysis were summarized in Table 2. All 15 studies (2699 patients) provided data on operative time. Meta-analysis of the operation time showed no statistically significant differences between the two groups (WMD = 0.38, 95% CI–10.43 –11.18, *P* = 0.95) (Figure 2).

**Table 2:** Pooled short-term outcomes of meta-analysis

Outcomes	Number of Study	Sample size		Heterogeneity (*P*, I^2^)	Overall effect size	95% CI of overall effect	*P* value
		LADG	TLDG				
**Operation time** (min)	20	2398	1811	< 0.01, 96%	WMD = 0.38	–10.43 to 11.18	0.95
**Blood loss** (ml)	18	1001783		< 0.01, 56%	WMD = 19.24	10.26 to 28.22	< 0.01
**Retrieved lymph nodes**	14	13651032		0.13, 31%	WMD = –0.99	–2.10 to 0.12	0.08
**Time to first flatus** (days)	10	885717		< 0.01, 95%	WMD = 0.27	–0.07 to 0.61	0.11
**Time to liquid diet** (days)	11	915767		< 0.01, 91%	WMD = 0.41	0.14 to 0.69	< 0.01
**Hospital stay** (days)	19	2328	1734	< 0.01, 78%	WMD = 0.72	0.31 to 1.13	< 0.01
**Overall complications**	13	14101077		0.78, 0%	WMD = 1.16	0.91 to 1.48	0.24

TLDG: Totally laparoscopic distal gastrostomy; LADG: laparoscopic-assisted distal gastrostomy; RR: risk ratio; WMD: weighted mean difference.

### Blood loss

**Figure 3:**
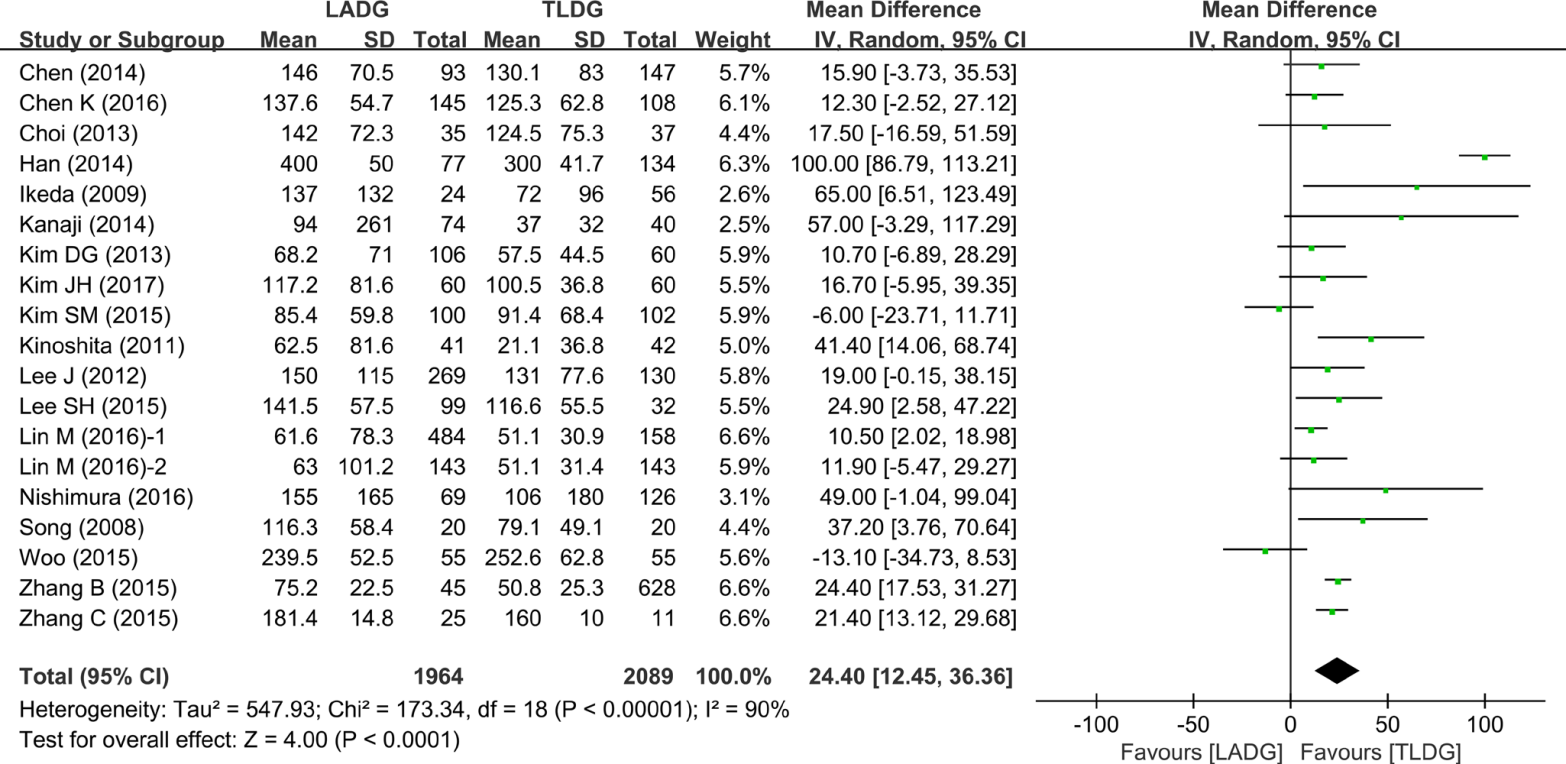
Meta-analysis of the pooled data: intraoperative blood loss

13 studies (1784 patients) provided data on amount of bleeding. Intraoperative bleeding was significantly reduced in the TLDG group (WMD = 24.4, 95% CI 12.45 –36.36, *P* < 0.0001) (Figure 3).

### Number of retrieved lymph nodes

**Figure 4:**
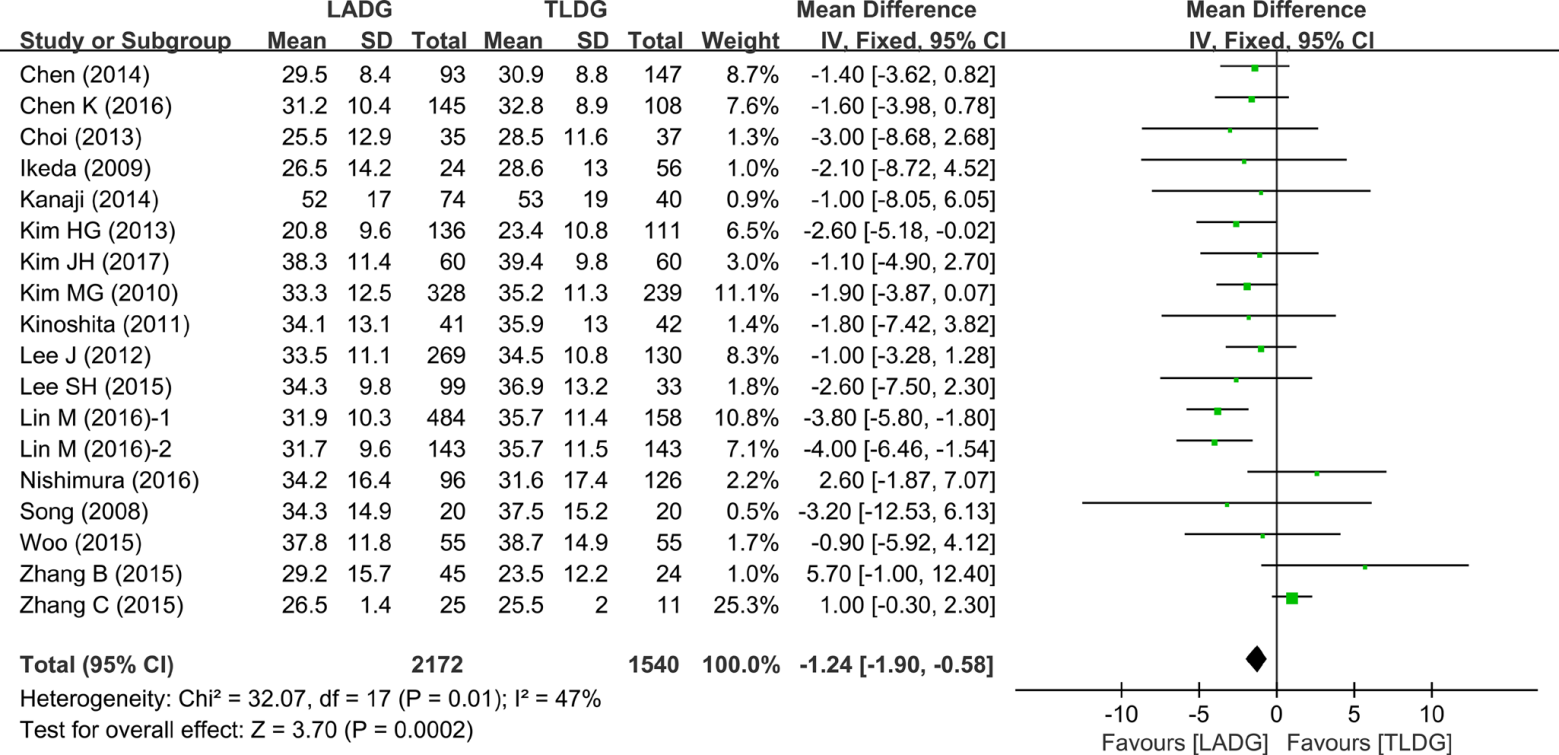
Meta-analysis of the pooled data: retrieved lymph nodes

The number of retrieved lymph nodes in LADG and TLDG was measured in 14 studies (2397 patients). There were more lymph nodes retrieved in the TLDG group (WMD = –1.24, 95% CI –1.90 to–0.58, *P* = 0.0002) (Figure 4).

### Time to liquid diet (days)

**Figure 5:**
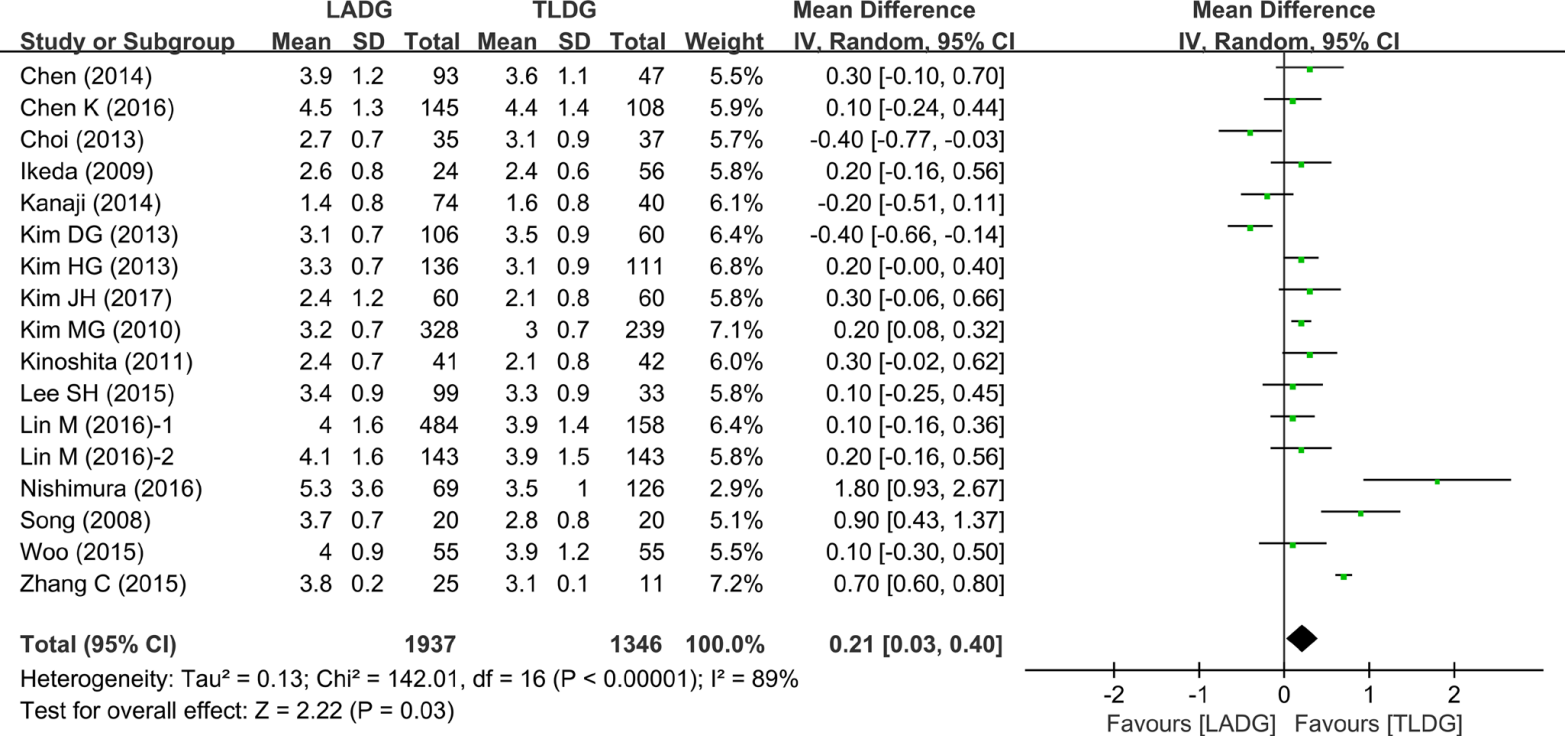
Meta-analysis of the pooled data: time to liquid diet (days)

11 studies (1245 patients) provided data on the time to first liquid. This was significantly shorter after TLDG than after LADG (WMD = 0.21, 95% CI 0.03–0.40, *P* = 0.03) (Figure 5).

### Postoperative hospital stay

**Figure 6:**
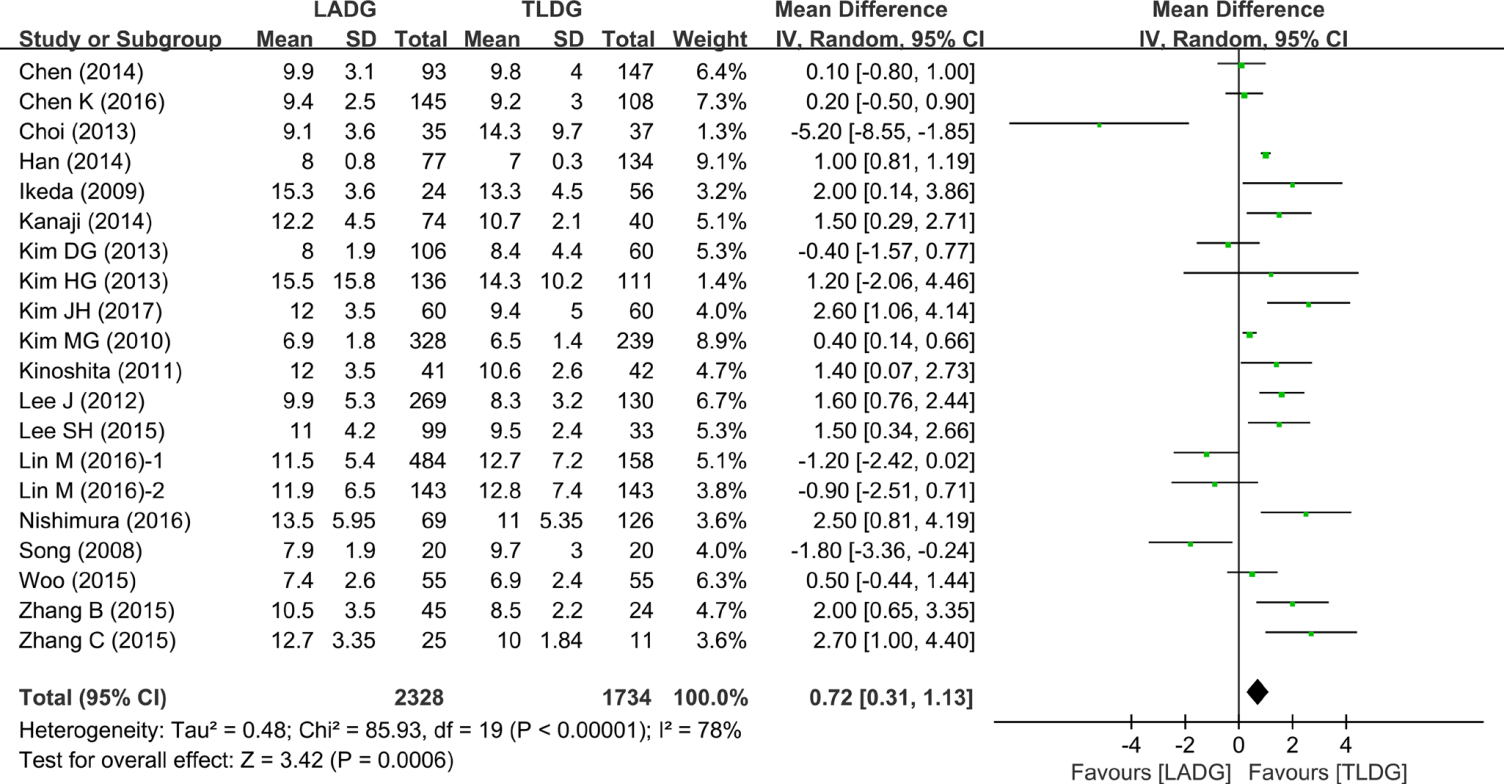
Meta-analysis of the pooled data: postoperative hospital stay

The number of days spent in hospital was compared in the 12 studies (2278 patients). Pooling the results, postoperative hospital stay was shorter after TLDG than after LADG (WMD = 0.72, 95% CI 0.31–1.13, *P* = 0.0006) (Figure 6).

### Postoperative complications

**Figure 7:**
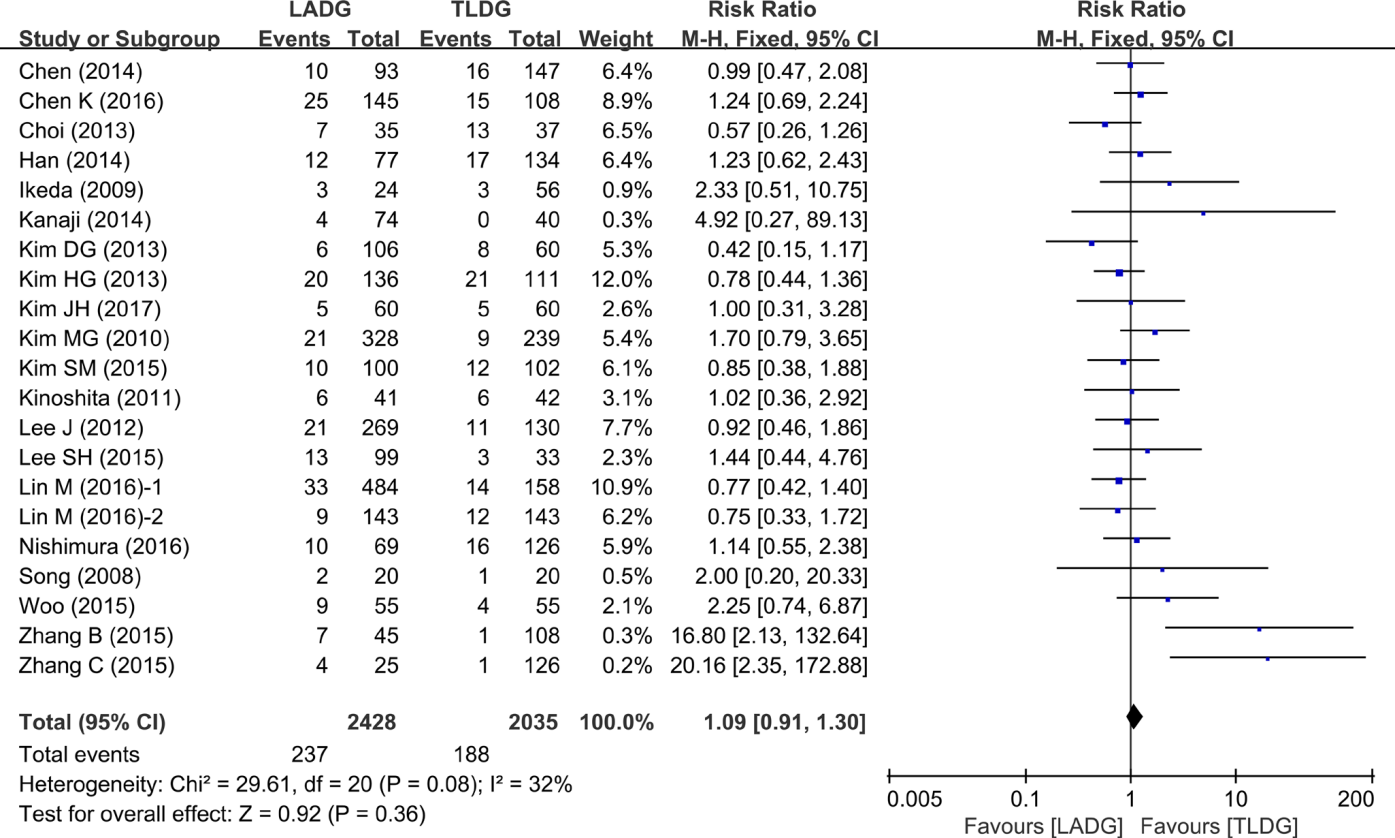
Meta-analysis of the pooled data: overall complications

**Figure 8:**
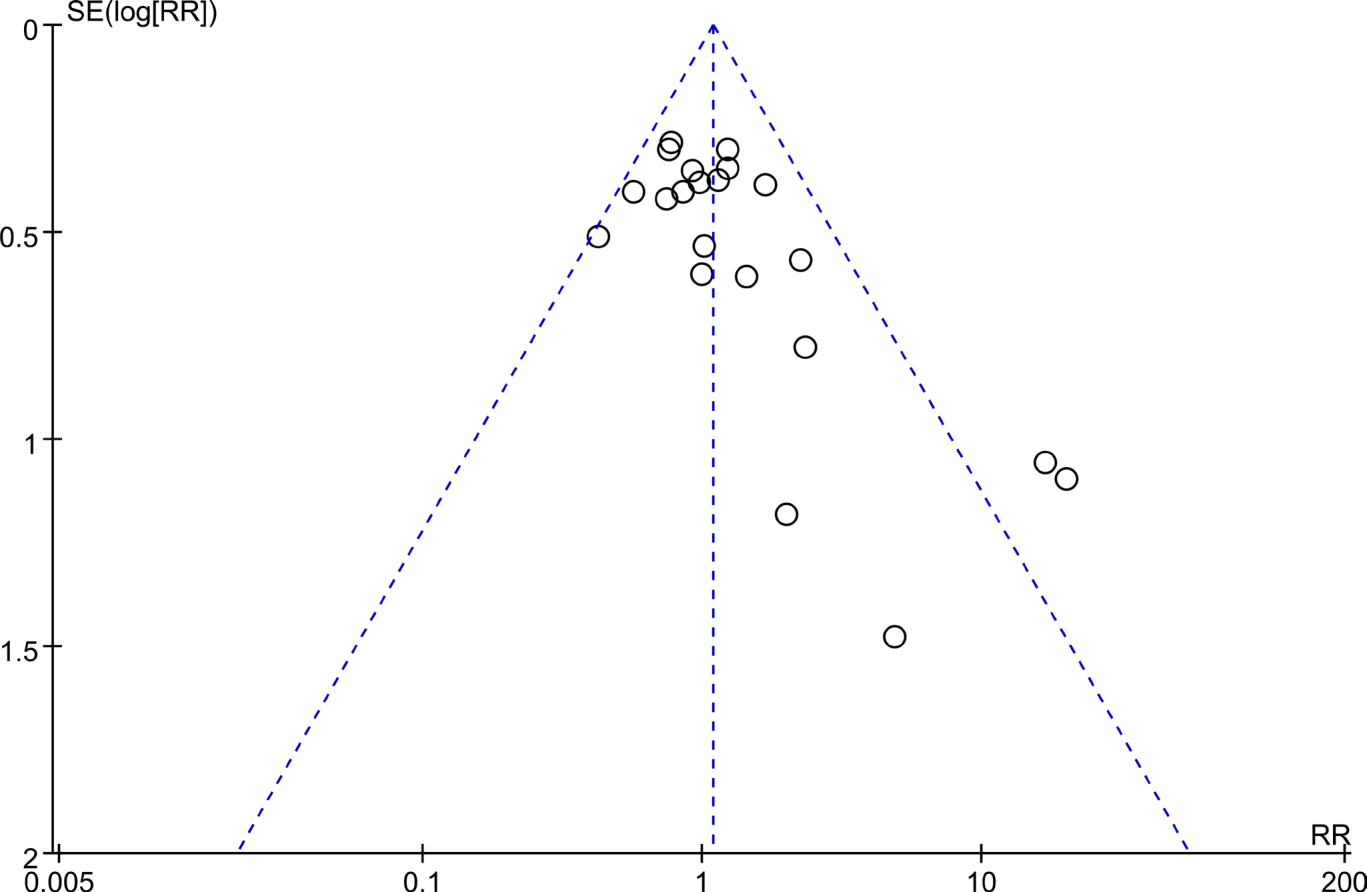
Funnel plots of the overall postoperative complications

13 studies (2487 patients) compared complications between TLDG and LADG. There was no statistically significant difference between the two groups (RR = 1.09, 95% CI 0.91–1.30, *P* = 0.36) (Figure 7). Visual inspection of the funnel plot revealed symmetry, indicating no serious publication bias (Figure 8).

## DISCUSSION

In the present meta-analysis, we found TLDG has several advantages. The overall conclusion revealed that TLDG has better operative and postoperative clinical outcomes such as less intraoperative blood loss, faster liquid diet, shorter postoperative hospital stay and more lymph nodes retrieved. But the incidence of postoperative complications (infection, obstruction and delayed gastric emptying and so on) and operative time were similar between the two groups.

LADG for the treatment of gastric cancer has undergone rapid development and gained popularity last decades. And the safety and therapeutic effect of LADG has been confirmed. Compared to the traditional open surgery, LADG can achieve better cosmesis, less intraoperative blood loss, shorter hospital stay, and better postoperative quality of life. And another surgical version is TLDG. The TLDG was first conceptualized and for the treatment of peptic ulcers in 1992 [29]. In 1996, TLDG was first applied to treat gastric cancer [30]. The most different procedures between the TLDG and LADG should be resection of the stomach and alimentary tract reconstruction. During LADG, the surgeon usually performs the most of procedures in laparoscopy except for the resection of the stomach and alimentary tract reconstruction through a small abdominal wall incision. And the structures around the anastomosis is likely to be injured due to the minilaparotomy, which made the surgeon under a limited working space, especially in an obese patient [31]. By contrast, TLDG can now be completed laparoscopically with the advancements of laparoscopic instruments and the accumulation of operative experience. That is, the entire procedure is observable in laparoscopy. Furthermore, unnecessary manipulations and the incision made on the epigastrium can be avoided. Thus, TLDG is considered less invasive than LADG. A few studies have described the benefits of intracorporeal anastomosis, such as small wound size and early bowel recovery [32]. Since the lack of support from large-scale randomized controlled studies (RCTs), the safety and benefit of TLDG surgery are still not well proven. To obtain a more reliable conclusion about the safety and benefit of TLDG, the research on the existing relevant data of TLDG-LADG comparative studies was conducted by using a meta-analysis.

Given the difficulty of reconstructing the digestive tract in laparoscopy, some surgeons are worried that TLDG may lead to prolonged operative time. Interestingly, the study conducted by Lee et al. [12], even revealed that the operation time of the TLDG group was shorter than the LADG group. And the present meta-analysis showed no significantly statistic difference between these two groups. This may mainly result from the use of the laparoscopic stapler instead of the laparoscopic suturing technique for Billroth I and Billroth II anastomoses. Compared to LADG, TLDG requires more skill with laparoscopic techniques. A surgeon should be well trained and with sufficient experience in laparoscopy before performing TLDG. So, TLDG was always performed during the late period of the surgeon’s experience and LADG performed during the early period. In addition, TLDG avoids the minilaparotomy in epigastrium, thus saving the time for cutting and suturing of the incision.

In our data, the intraoperative blood loss was reduced in the TLDG group. We thought there are several reasons causing the present result. Usually, it is well known that the incision at the epigastrium required by LADG is bigger than that in TLDG at the umbilicus, which would lead to more blood loss. Moreover, excessive stretch for the gastric stump out of the abdominal cavity and the anastomosis through the minilaparotomy by hand manipulation may injure the surrounding tissues and the anastomosis itself. That is one of the main reasons why LADG causes more blood loss. However, this result should be interpreted cautiously. Because the heterogeneity (I^2^ = 90%, *p* < 0.0001) in the studies was high, in spite of the pooled data of intraoperative blood loss have a significantly statistical difference.

The present meta-analysis revealed that the number of lymph nodes retrieved in the TLDG group is more than that in LADG. As is known to all, the number of the harvest lymph nodes is a critical measure of success in laparoscopic surgery for malignant tumour of the stomach. Since the two groups have the similar approaches of retrieving lymph nodes, the reasons why TLDG and LADG have different numbers of lymph nodes depend mainly on the level of surgical skills. Considering the difficulties of TLDG, it was always performed during the late period of the surgeon's experience and LADG performed during the early period. Without the data of how the specimens were processed in the studies, we can't draw a definite conclusion.

As for the evaluation of the postoperative recovery measurements, we analysis the pooled data of time to liquid diet, postoperative hospital stay and operative complications. Compared to LADG, TLDG was associated with faster liquid diet and shorter postoperative hospital stay. Meantime, our study showed that there was no significant difference in the overall postoperative complications between the TLDG group and the LADG group. TLDG has been shown to reduce the incidence of touching and tension during anastomosis, which would lead to earlier bowel function recovery and faster liquid diet. Small incisions, earlier bowel function recovery, faster liquid diet and similar incidence of postoperative complications compared with LADG contribute to shorter post-hospital stay in the TLDG group. The conclusion we has drawn from the present analysis appears to be that TLDG is a less invasive procedure than LADG.

There are some limitations existing in our study. Due to the lack of high quality randomized controlled trials (RCTs), the materials of our study almost consisted of the observational clinical studies, so it was almost inevitable to bias the final outcome in the non-RCTs. Second, some research results might not be published, especially the gray literatures containing negative results, which inevitably caused publication bias. Finally, the different disease condition and surgeon’ experience in each study was inconsistent, which would lead to a degree of clinical heterogeneity.

In summary, the present meta-analysis indicates that TLDG might be a feasible, safe, beneficial surgical method for the treatment of gastric cancer. The analysis also shows that TLDG was favorable in terms of less intraoperative blood loss, faster liquid diet and shorter postoperative hospital stay and more lymph nodes retrieved, compared to LADG. But there are no significant difference in the incidence of postoperative complications and operative time. Maybe large randomized controlled trials and more methodologically high-quality comparative studies are required to adequately evaluate the superiority of TLDG for gastric cancer.

## MATERIALS AND METHODS

We performed a comprehensive search of English-language publications listed in the electronic database PubMed, Web of Science, and EMBASE. All references of retrieved articles were reviewed to identify all the potential studies. The search terms were as follows: ‘gastric cancer’, ‘gastric neoplasms’, ‘gastric adenocarcinoma’, ‘laparoscopic’, ‘laparoscopy’, ‘gastrectomy’, ‘totally’ and ‘intracorporeal’, ‘extracorporeal’. The last search was conducted on June, 2017.

### Inclusion and exclusion criteria

For this meta-analysis, the inclusion and exclusion criteria were as follows: (1) Including all articles comparing LADG and TLDG for gastric cancer; (2) Clear case selection criteria and report on at least the following information: the number of cases, surgical methods and standard deviation; (3) And if there was overlap between authors or centers, the higher quality or more recent literature was selected; (4) Abstracts, letters, editorials, expert opinions, reviews without original data, case reports, and studies without control groups were excluded. All the eligible manuscripts were carefully scrutinized by two independent authors. To reach a consensus, disagreements on the conflicting results were resolved between the two authors.

### Data extraction

To minimize bias and improve the reliability, two authors independently collected information from each study. The extracted data included: author, study period, number of patients, operation time, blood loss, number of retrieved lymph nodes, time to liquid diet, length of postoperative hospital stay and surgery-related complications. Discrepancies between the two reviewers were resolved by discussion and consensus.

### Statistical analysis

This meta-analysis was performed using the Review Manager (RevMan) software, version 5.3. We analyzed the dichotomous variables by estimating the odds ratios (OR) with a 95% confidence interval (95% CI), and continuous variables were analyzed using weighted mean difference (WMD) with a 95% CI. Statistical heterogeneity, which indicated between-study variance, was evaluated according to the Higgins I^2^ statistic. A probability value of *P* < 0.05 and/or I^2^ > 50% indicated significant heterogeneity, and a random-effects model was used depending on the heterogeneity analysis. Otherwise, a fixed-effect model was applied. Potential publication bias was determined by conducting an informal visual inspection of funnel plots based on the complications.
